# Health Care Utilization and Symptom Severity in Ghanaian Children – a Cross-Sectional Study

**DOI:** 10.1371/journal.pone.0080598

**Published:** 2013-11-14

**Authors:** Ralf Krumkamp, Nimako Sarpong, Benno Kreuels, Lutz Ehlkes, Wibke Loag, Norbert Georg Schwarz, Hajo Zeeb, Yaw Adu-Sarkodie, Jürgen May

**Affiliations:** 1 Bernhard Nocht Institute for Tropical Medicine, Hamburg, Germany; 2 Kumasi Centre for Collaborative Research in Tropical Medicine, Kumasi, Ghana; 3 Section for Tropical Medicine, 1st Medical Department, University Medical Centre Hamburg-Eppendorf, Hamburg, Germany; 4 Bremer Institute for Prevention Research and Social Medicine, University of Bremen, Bremen, Germany; 5 School of Medical Sciences, Kwame Nkrumah University of Science and Technology, Kumasi, Ghana; National Institute of Medical Research, United Kingdom

## Abstract

The aim of this study was to identify factors influencing health care utilization behavior for children with mild or severe disease symptoms in rural Ghana. Between March and September 2008 a cross-sectional health care utilization survey was conducted and 8,715 caregivers were interviewed regarding their intended behavior in case their children had mild or severe fever or diarrhea. To show associations between hospital attendance and further independent factors (e.g. travel distance or socio-economic status) prevalence ratios were calculated for the four disease symptoms. A Poisson regression model was used to control for potential confounding. Frequency of hospital attendance decreased constantly with increasing distance to the health facility. Being enrolled in the national health insurance scheme increased the intention to attend a hospital. The effect of the other factors diminished in the Poisson regression if modeled together with travel distance. The observed associations weakened with increasing severity of symptoms, which indicates that barriers to visit a hospital are less important if children experience a more serious illness. As shown in other studies, travel distance to a health care provider had the strongest effect on health care utilization. Studies to identify local barriers to access health care services are important to inform health policy making as they identify deprived populations with low access to health services and to early treatment.

## Introduction

The Millennium Development Goal (MDG) 4 stipulates that, between 1990 and 2015, the mortality rate of children aged younger than five years should be reduced by two thirds. Recent estimates suggest that childhood mortality is decreasing in the developing world but still not fast enough to reach MDG 4 [1]. Achievements to reduce childhood deaths differ strongly by geographical region and especially sub-Saharan-Africa is still burdened by high infant and child mortality rates [2,3]. Accessible professional health services make early treatment possible, thus reducing serious health consequences. Access to and provision of health services vary strongly between geographical regions, and even within countries [4,5]. Several studies in developing countries identified barriers to health care utilization (HCU) such as low socio-economic status, travel distance to service providers, poor knowledge about diseases or the perceived quality of the health care provider [6–9]. Disease severity in contrast, increased the likelihood of seeking professional medical treatment [7]. However, less is known about how HCU barriers differ between symptoms considering differences in symptom severity. 

The aim of this study was to identify factors influencing health care utilization behavior for children with mild and severe fever or diarrhea in the Asante Akim North District, a typical rural area in Ghana. 

## Methods

Between March and September 2008 a cross-sectional health care utilization survey was conducted within the Asante Akim North District, Ghana. 

The study and the informed consent procedure was approved by the Committee on Human Research Publications and Ethics of the College of Health Sciences, School of Medical Sciences, Kwame Nkrumah University of Science and Technology, Kumasi, Ghana.

### Study area

The survey was conducted in two overlapping hospital catchment areas. The Agogo Presbyterian Hospital (APH) is a district hospital with about 250 patient beds. Among other facilities, it has a separate children’s Outpatient Department (OPD) and a pediatric ward. The Konongo-Odumasi Hospital (KOH) is the government district hospital located in the district capital, with 42 patient beds of which eight belong to the children’s ward. It has a general OPD for both children and adults. The study area has a rural character with an estimated population of 170,000 inhabitants spread over 1,160 square kilometers. The climate is tropical and the region is mainly covered by secondary rain forest and cultivated land.

### Study population

Study households were randomly selected using a probability proportional to size cluster-sampling method. Sampling frame data was obtained from the Asante Akim North District Planning Office. Families had to live in the study area for at least six months in order to be selected. In a first step, local authorities (i.e. chiefs, assemblymen, opinion leaders) of the communities throughout the study area were approached to introduce the research team and to explain the purpose of the study carefully. When permission was given to conduct interviews residents were informed about the study highlighting that participation was voluntary. Appointments with the selected study households were made. Finally, field workers visited families at their home and interviewed caregivers of children aged up to twelve years after verbal informed consent was given. Consent was given in the presence of a witness and documented for each participant on a study form. Verbal consent was chosen because no samples or individual health data were collected. Study participants were free to terminate the interview at any time. One person refused to participate. Interviewees were asked questions regarding their health care seeking behavior in case their children showed a particular disease symptom, suggesting different severity stages of a febrile illness or a gastrointestinal infection. Symptom duration was used to differentiate between perceived disease severity. Symptoms considered for the study were (i) acute fever, (ii) fever for three days, (iii) acute diarrhea, and (iv) diarrhea for one week. In addition, demographic data, information about health behavior, and socio-economic data were collected, and the GPS (Global Positioning System) coordinates of the households were recorded. 

The resulting dataset has previously been analyzed to identify the actual catchment population of APH in order to estimate incidences for childhood diseases [10–12], as well as to describe the association between socio-economic status and enrolment in the national health insurance program [13].

### Study design

Participants could choose whether they would seek help at APH, KOH or another hospital, or whether they would visit a local healer, a pharmacy or treat their children at home. Interviewees who replied to visit APH or KOH if their children showed a particular disease symptom were classified as a hospital attendee. Non-attendees were defined as interviewees who would visit a local healer, a pharmacy, or treat their children at home. Due to the single choice character of the questioning the preferred hospital of non-attendees is unknown. To be included into the analyses, study participants had to belong to the respective hospital catchment groups of APH or KOH. Some study participants reported not to visit a hospital in case their child had acute fever or diarrhea, however they would visit a hospital other than APH or KOH if the respective more severe disease symptoms (i.e. fever for three days or diarrhea for one week) occurred. These individuals were removed from the analyses. Thus, different sets of study participants were selected, resulting in varying study sizes for different symptom groups. 

### Geographical data and distance measurements

The GPS coordinates of the households were mapped using ArcGIS 10 (Esri, Redlands, CA, USA). To calculate travel distances between the households and APH or KOH, a road map shapefile was acquired from DIVA-GIS (http://www.diva-gis.org/). In this shapefile, main streets and roads were recorded, however, smaller paths were not. Therefore, the air-line distance from each household to the nearest road, and the subsequent road distance to the hospitals were calculated with the Network Analyst extension (Esri, Redlands, CA, USA). The sum of path and road distance was used to estimate the individual travel distance to each hospital. Some study participants had an unreasonably long travel distance, especially when missing road information led to an unlikely combination of roads to travel. These study participants were identified via the distance ratio of travel distance over air-line distance. The travel distance of those study participants with a distance ratio above the 95^th^ percentile were replaced using their air-line distance to the next hospital multiplied by the mean distance ratio in order to account for the longer road journey. For attendees the distance to their reported hospital was used in the analysis. For non-attendees the distance to their nearest hospital was used since no information about their preferred hospital was available. Consequently, only attendees that would attend their nearest hospital could be considered in the studies, as this restriction applied to non-attendees as well.

### Data analysis

A relative socio-economic status (SES) score was constructed with a principal component analysis (PCA) [14]. Dichotomous asset and education variables were used to set up the score, namely living in a brick house, in-house toilet available, cooking inside, domestic tap-water available, electricity available, owning a fridge, difficulty to manage income, literacy of the mother, and literacy of the father. The constructed linear score was categorized to quartiles to generate the four SES-groups poor, low, moderate, and high. 

To describe variable distributions the mean with the respective standard deviation (SD) was calculated for normally distributed continuous variables, or the median with the respective interquartile range (IQR) for the non-normally distributed continuous variables. Categorical variables were described showing the absolute frequency with the corresponding percent. Outcome measure of the study was the prevalence of hospital attendance, which is the number of attendees over the sum of attendees and non-attendees. To show associations between symptom-related HCU and travel distance, the prevalence of hospital attendance within a moving window along the sorted distance variable was calculated (moving prevalence). Each window contained 100 individuals and moved individual-wise through the travel distance. This method is adapted from the moving average approach to show smoothed changing proportions of a dichotomous variable along a sorted linear variable. Crude prevalence ratios (PR) and their corresponding 95%-confidence intervals (CI) were calculated via a cross-table to show associations between two dichotomous variables and effect differences along a second categorical variable were assessed by stratification. Finally, a Poisson regression analysis (with a robust error variance [15,16]) was performed to show interactions between independent variables and to control for potential confounding. The model was set up based on prior knowledge, including variables previously reported to be associated with HCU. To show how associations change between the different symptoms, the same set of independent variables was used to construct regression models for each symptom group. Observations with missing values in independent variables were ignored in the particular analyses. Thus, the denominator for some statistics may differ. Statistical analyses were performed using STATA 12 (StataCorp LP, College Station, USA).

## Results

### Study population

Throughout the study area 8,715 interviews were conducted in 138 villages. Village size varied from 18 to 15,383 inhabitants with a median size of 299 inhabitants (IQR 139-621). Almost all interviews were conducted with females (8,643; 99.2%) with a mean age of 36.2 years (SD 12.6). 3,321 interviewees (38.1%) reported to be enrolled in the national health insurance (NHI) scheme. Household size varied from two up to 32 people. The mean number of people per household was 6.0 (SD 2.8). The number of children per household varied between one and 25, with a mean number of 3.2 (SD 2.0) children. Living conditions differed strongly between families. Table 1 gives an overview of study participants’ characteristics and the distribution of the variables used to model the SES-score. 

**Table 1:** Characteristics of the interviewees (N = 8,715), Asante Akim North District, Ghana, 2008.

**Variable**	**frequency (%**)** or mean** (**SD** ^§^)
Female interviewees	8,643 (99.2)
Age of interviewees, mean years	36.2 (SD 12.6)
Enrolled in National Health Insurance programme	3,321 (38.1)
People per household, mean number	6.0 (SD 2.8)
Children per household, mean number	3.2 (SD 2.0)
Illiteracy mother	6,101 (70.1)
Illiteracy father	3,152 (36.2)
Living in brick or stone houses	6,097 (70.0)
Domestic tab-water	6,797 (78.0)
In-house electricity	4,459 (53.5)
In-house cooking facilities	5,707 (65.5)
In-house toilets	2,846 (32.7)
Owning refrigerator	1,190 (13.7)
Difficulty to manage income	5,519 (63.3)

§ SD, Standard Deviation

According to the study participants, alternative health care facilities (HCF) were available in 27 of 138 villages, within which 4,631 (53.1%) of the interviewees lived. 2,618 (30.0%) reported to have some kind of medication at home. Of the children, 3,500 (40.2%) were born at home and 5,044 (57.9%) were born in a health facility. The place of birth for the remaining children was unknown. 519 (6.0%) of the interviewees reported to have never attended a hospital so far. Of the interviewees, 2,928 (33.6%) attended APH and 1,795 (20.6%) attended KOH at their last hospital visit. For 3,501 (40.2%) interviewees APH and for 5,214 (59.8%) KOH was the nearest hospital. Interviewees lived up to 72.9 km away from their nearest hospital, with a median travel distance of 12.9 km (IQR 3.1-23.8 km). 

### Hospital attendance

Different study groups comprising each around 50% of all observations were constructed for the symptom dependent HCU analyses. Table 2 shows the number of study participants who would attend a hospital for the different symptoms for all interviewees and the prevalence of hospital attendance within each symptom groups. A higher proportion of parents reported to visit a hospital in case their children had diarrhea compared to fever. For both fever for three days and diarrhea for one week, the prevalence of hospital attendance was more than doubled compared to the respective milder symptoms. 

**Table 2:** Disease symptoms and number of intended hospital visits within the whole group, the study size of the symptom-based study groups, and the intended hospital visits within the constructed symptom-based study groups, Asante Akim North District, Ghana, 2008.

**Symptoms**	**Hospital attendance within the whole group^a^**	**Sample size of the symptom-based study group^b^,^c^**	**Hospital attendance within the symptom-based study group^b^**
Acute fever	3,006 (34.5)	4,437 (48.3)	1,330 (30.0)
Fever for three days	7,944 (91.2)	4,320 (47.1)	3,570 (82.6)
Acute diarrhoea	3,952 (45.3)	4,285 (46.7)	1,738 (40.6)
Diarrhoea for one week	8,241 (94.5)	4,177 (45.5)	3,714 (88.9)

^a^ whole study group, comprising all interviewees (N = 8,715)

^b^ constructed study groups, comprising attendees and non-attendees for the respective disease symptoms

^c^ per cent of the whole study group

The binary associations between hospital attendance and potential influencing factors for all symptom groups are shown in Table 3. For all symptoms, the reported hospital attendance decreased with increasing travel distance, yet this trend was less marked for the more severe symptoms. The PR for distance suggests minor effects, however one has to consider that the prevalence change per 1 km unit was modeled. The PR of 0.98 (CI 0.98-0.99) as calculated for fever for three days, can be extrapolated to a respective PR of 0.85 (CI 0.83-0.86), 0.72 (CI 0.69-0.75), 0.61 (CI 0.57-0.65), and 0.51 (CI 0.47-0.56) for 10 km, 20 km, 30 km, and 40 km travel distance. Having a functional health care facility in the community did not influence the decision to attend a hospital. SES had the same effect for the milder symptoms, with the PR for clinic attendance increasing slightly towards the higher SES quintiles. No association between SES and hospital attendance was observed for more severe symptoms. Similar associations were seen for enrolment in the NHI scheme.

**Table 3:** Bivariate associations (prevalence ratios with 95%-confidence interval) between hospital attendance and explanatory variables for the different disease symptoms, Asante Akim North District, Ghana, 2008.

**Regressant**	**Distance** (**1 km unit**)^a^	**Alternative HCF** ^b$^	**SES-groups (Q1 vs. Q2-Q4**)^c$^	**Health insurance** ^b^
**Acute fever**	0.96 (0.95-0.96)	1.0 (0.9-1.2)	Q1: ref.	1.4 (1.3-1.6)
			Q2: 1.4 (1.2-1.7)	
			Q3: 1.6 (1.4-1.9)	
			Q4: 1.7 (1.5-1.9)	
**Fever for three days**	0.98 (0.98-0.99)	1.0 (0.9-1.0)	Q1: ref.	1.1 (1.1-1.2)
			Q2: 1.1 (1.1-1.2)	
			Q3: 1.2 (1.1-1.3)	
			Q4: 1.2 (1.2-1.3)	
**Acute diarrhoea**	0.97 (0.96-0.98)	1.1 (1.1-1.3)	Q1: ref.	1.4 (1.3-1.5)
			Q2: 1.4 (1.2-1.5)	
			Q3: 1.5 (1.3-1.7)	
			Q4: 1.6 (1.4-1.8)	
**Diarrhoea for one week**	0.99 (0.99-0.99)	1.0 (1.0-1.0)	Q1: ref.	1.1 (1.0-1.1)
			Q2: 1.1 (1.1-1.2)	
			Q3: 1.1 (1.1-1.2)	
			Q4: 1.1 (1.1-1.2)	

^a^ calculated via Poisson regression, two decimal places shown to detail smaller effects

^b^ calculated via cross-tables

^c^ calculated via Poisson regression

^$^ HCF, health care facility; SES, socio-economic status; Q1-Q4, first quintile – fourth quintile

**Figure 1:**
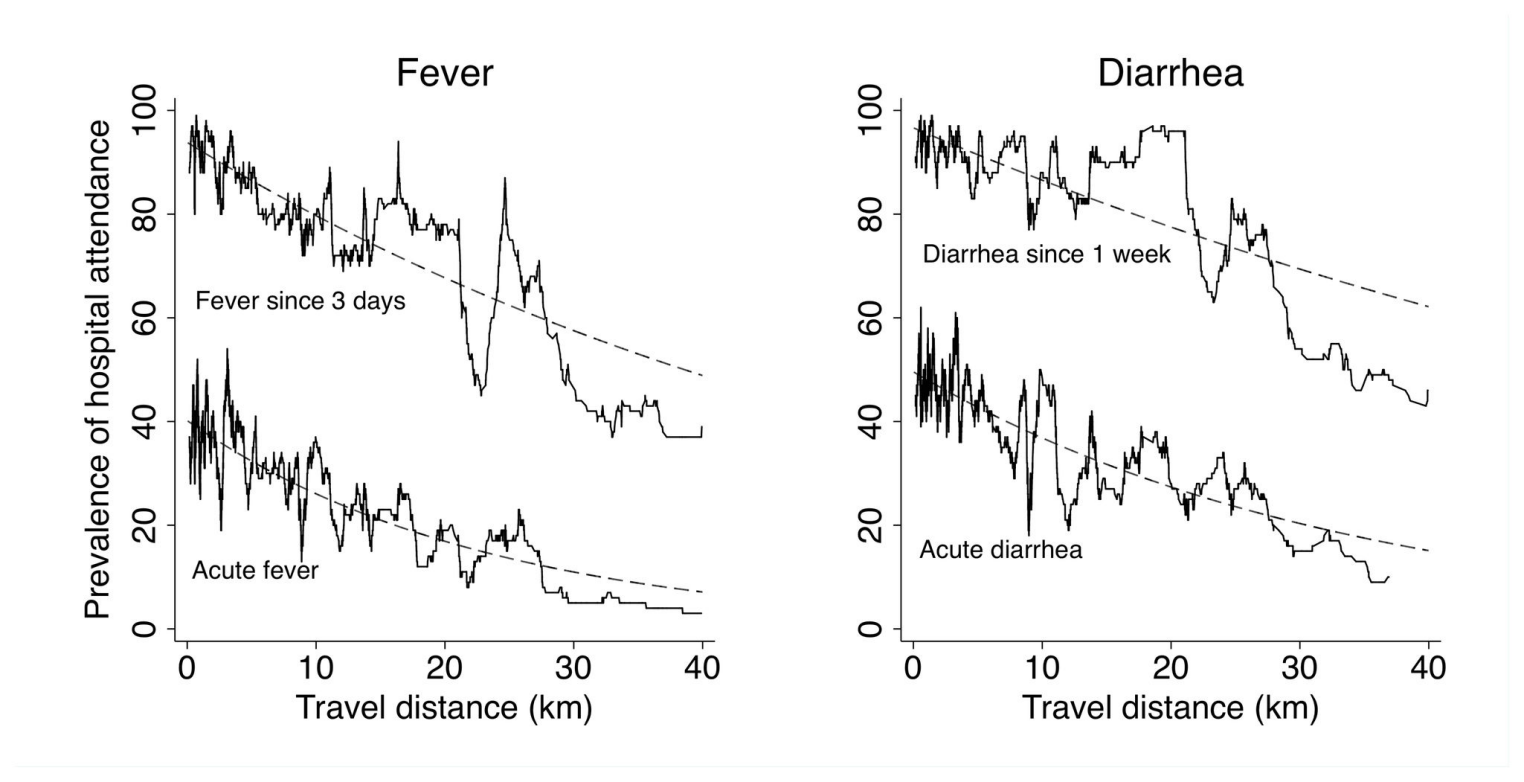
Observed prevalence (solid line) and predicted prevalence (dashed line) of intended hospital attendance by symptom along the travel distance to the nearest hospitals, Asante Akim North District, Ghana, 2008.

The moving prevalence and the predicted prevalence (based on the Poisson regression analysis shown in Table 3) of hospital attendance along the travel distance is shown in Figure 1. The prevalence of hospital attendance continuously decreased with increasing distance between household and nearest hospital for all disease symptoms. However, the observed variability indicated that factors along the travel distance had an additional effect on the decision to visit a hospital. Some prevalence peaks occurred at distances where larger villages are located. 

### Multivariate Poisson regression model

Among the explanatory variables associations were observed between distance to hospital and SES group, as well as between being health insured and SES group. Individuals with a higher SES tended to live closer to a hospital, and individuals with a higher SES were more likely to be enrolled in the national health insurance scheme. To adjust for potential confounding and assess potential interaction between explanatory variables, multivariate regression models were built for each symptom. The regression models for all symptoms showed strong confounding and most variables lost their associations if modeled together with travel distance. In the models for mild fever and mild diarrhea, distance to hospital and health insurance were the only variables that showed a significant influence on hospital attendance (Table 4). 

**Table 4:** Poisson regression models of the relationship between hospital attendance and explanatory variables for the different disease symptoms, Asante Akim North District, Ghana, 2008.

**Regressant**	**Distance (1 km unit)^a^**	**Alternative HCF** ^$^	**SES-groups (Q1 vs. Q2-Q4**)^$^	**Health insured**
**Acute fever**	0.96 (0.95-0.97)	1.0 (0.9-1.1)	Q1: ref.	1.3 (1.2-1.4)
			Q2: 1.1 (0.9-1.3)	
			Q3: 1.1 (0.9-1.3)	
			Q4: 1.1 (0.9-1.2)	
**Fever for three days**	0.98 (0.98-0.99)	0.9 (0.9-1.0)	Q1: ref.	1.1 (1.1-1.1)
			Q2: 1.0 (1.0-1.1)	
			Q3: 1.0 (1.0-1.1)	
			Q4: 1.0 (1.0-1.1)	
**Acute diarrhoea**	0.97 (0.97-0.98)	1.1 (1.0-1.2)	Q1: ref.	1.3 (1.2-1.4)
			Q2 1.1 (1.0-1.3)	
			Q3: 1.1 (1.0-1.3)	
			Q4: 1.1 (1.0-1.3)	
**Diarrhoea for one week**	0.99 (0.99-0.99)	1.0 (0.9-1.0)	Q1: ref.	1.1 (1.0-1.1)
			Q2: 1.0 (1.0-1.1)	
			Q3: 1.0 (1.0-1.1)	
			Q4: 1.0 (1.0-1.0)	

HCF, health care facility; SES, socio-economic status; Q1-Q4, first quintile-fourth quintile

a two decimal places shown to detail smaller effects

## Discussion

The aim of the current study was to identify factors influencing a caregivers’ decision to seek help in rural Ghana in case their children showed a particular disease symptom, taking varying disease severity into account. For the disease symptoms studied (i.e. acute fever, fever for three days, acute diarrhea, and diarrhea for one week) the intention to visit a hospital decreased constantly with increasing travel distance. Being enrolled in the NHI scheme increased the willingness to attend a hospital. The effect of SES and availability of a health facility in the home village diminished in the Poisson regression if modeled together with travel distance. The observed associations weakened with increasing symptom severity, which indicates that barriers to visit a clinic are less predominant if children experience a more serious illness. 

Travel distance shows the strongest association with clinic attendance. Its effect has been shown in a number of previous studies and is often referred to as distance-decay. For instance, studies showed these decay effects for children [17–19], and adults [19] using actual health seeking behavior data, and for children [20,21] using reported health seeking behavior. Studies from Ethiopia [22] and Burkina Faso [23] demonstrated that child mortality increases with distance to health facilities. In the current study higher willingness to attend a hospital was observed at distances where larger villages are located suggesting that interviewees from larger villages have easier access to the hospitals, thus weakening the distance-decay. We used the estimated travel distance, which does not consider actual travel time or further travel related efforts. So far, little is known about the association of HCU and travel dependent factors. A study from Malawi showed higher likelihood of HCU for villages with access to public transport [24], and another study from Pakistan reported that the availability of public transport as well as the frequency of the provided services are important to access remote HCFs [25]. It appears highly plausible that availability of transport plays an important role, but issues such as affordability also need to be considered. 

The current study does not show associations between SES and HCU after adjusting for travel distance. Associations shown in the bivariate analysis are confounded by travel distance, as interviewees living in remote rural areas tend to have a lower SES. Several studies have addressed the topic of SES and health seeking behavior and showed differing results across regions in the developing world. In Tanzania [26] and South Africa [27] higher SES groups had an increased likelihood of hospital attendance. Yet also in these studies, participants with a higher SES lived closer to the HCF, but the analyses were not controlled for potential confounders. In Uganda [28], Ghana [29], and Nigeria [30] individuals with lower SES utilize health services less often. Another study from Nigeria demonstrated that patients with a lower SES use professional malaria treatment less often but this association could not be confirmed if controlled for the quality of the service provider [31]. The cited studies as well as the current study illustrate the methodological challenges to untangle confounded associations between SES and HCU. 

In 2004 the National Health Insurance Scheme was implemented in Ghana. If insured, people receive free treatment for pre-defined health conditions. The insurance is social security and tax financed and had a coverage of 62% in 2009, with significant regional variations [32]. Evaluations showed that the NHI scheme improved the access to health care services but it is still fragmented [33] and less available for the poor [34]. In our study the enrolment in the NHI scheme increased the likelihood of HCU by approximately 30% (for the milder symptoms). This finding is supported by another study from Ghana, which analyzed the use of professional health care services for children with fever [35]. A universal health insurance, providing free access to professional treatment, could bridge the gap especially to poorer groups within the population. 

Our data suggest that more efforts are mounted to overcome HCU barriers if the health conditions are perceived to be more serious. Disease severity has already been reported to be an important driver for treatment seeking in other studies [29,36]. Nevertheless, caregivers often underestimate the seriousness of diseases [37]. Easy access to professional medical treatment is crucial to prevent aggravation of diseases, and to reduce disease complications and mortality. 

A limitation of the current study is that the analysis is based on self-predicted intended behavior of caregivers in case their children showed particular disease symptoms. This approach enables the analysis of health behavior with regard to different disease symptoms. However, we have to rely on reported behavior, which is prone to reporting bias. Interviewees are likely to over-report hospital attendance finally leading to an overall higher prevalence of HCU. An alternative approach would be the assessment of the actions taken by parents during the last illness of their child. This would also allow to differentiate effects of age and gender, which could not be assessed with the current methodology. Especially age differences are reported in the literature. For younger children caretakers tend to seek professional health earlier and more frequent compared to the elder ones [17,19,29].

Cross-sectional study designs allow the calculation of PRs, which yield comparable results among study groups even with different frequencies of the study outcome. This is not the case if the odds ratio (OR) is calculated from studies based on a cross-sectional sampling approach. Here the rare-disease-assumption has to be fulfilled to estimate the risk ratio; otherwise the OR is likely to overestimate the effect of a risk ratio [38]. Because the frequency of disease symptoms varies among the established symptom-based study groups, it was crucial to use a comparable effect estimator and thus PRs were calculated and the corresponding Poisson regression was applied. 

Studies, like the current one, are informing public health policy makers to develop strategies to reach deprived population groups. Recent evaluation advocated this equity aspect in the provision of national health care services. Reaching most deprived population with less access to health services is a cost-effective approach to save lives in the developing world [39]. 

## References

[1] Lozano R, Wang H, Foreman KJ, Rajaratnam JK, Naghavi M et al. (2011) Progress towards Millennium Development Goals 4 and 5 on maternal and child mortality: an updated systematic analysis. Lancet 378: 1139-1165. doi:10.1016/S0140-6736(11)61337-8. PubMed: 21937100. 21937100

[2] Hill K, You D, Inoue M, Oestergaard MZ (2012) Child mortality estimation: accelerated progress in reducing global child mortality, 1990-2010. PLOS Med 9: e1001303 PubMed: 22952441. 22952441 10.1371/journal.pmed.1001303 PMC3429379

[3] Wang H, Dwyer-Lindgren L, Lofgren KT, Rajaratnam JK, Marcus JR et al. (2013) Age-specific and sex-specific mortality in 187 countries, 1970-2010: a systematic analysis for the Global Burden of Disease Study 2010. Lancet 380: 2071-2094. PubMed: 23245603. 10.1016/S0140-6736(12)61719-X 23245603

[4] Chopra M, Sharkey A, Dalmiya N, Anthony D, Binkin N (2012) Strategies to improve health coverage and narrow the equity gap in child survival, health, and nutrition. Lancet 380: 1331-1340. doi:10.1016/S0140-6736(12)61423-8. PubMed: 22999430. 22999430

[5] Rutherford ME, Mulholland K, Hill PC (2010) How access to health care relates to under-five mortality in sub-Saharan Africa: systematic review. Trop Med Int Health 15: 508-519. doi:10.1111/j.1365-3156.2010.02497.x. PubMed: 20345556. 20345556

[6] Peters DH, Garg A, Bloom G, Walker DG, Brieger WR et al. (2008) Poverty and access to health care in developing countries. Ann N Y Acad Sci 1136: 161-171. doi:10.1196/annals.1425.011. PubMed: 17954679. 17954679

[7] Chuma J, Abuya T, Memusi D, Juma E, Akhwale W et al. (2009) Reviewing the literature on access to prompt and effective malaria treatment in Kenya: implications for meeting the Abuja targets. Malar J 8: 243. doi:10.1186/1475-2875-8-243. PubMed: 19863788. 19863788 PMC2773788

[8] Kiwanuka SN, Ekirapa EK, Peterson S, Okui O, Rahman MH et al. (2008) Access to and utilisation of health services for the poor in Uganda: a systematic review of available evidence. Trans R Soc Trop Med Hyg 102: 1067-1074. doi:10.1016/j.trstmh.2008.04.023. PubMed: 18565559. 18565559

[9] Obrist B, Iteba N, Lengeler C, Makemba A, Mshana C et al. (2007) Access to health care in contexts of livelihood insecurity: a framework for analysis and action. PLOS Med 4: 1584-1588. PubMed: 17958467. 17958467 10.1371/journal.pmed.0040308 PMC2039761

[10] Marks F, Adu-Sarkodie Y, Hünger F, Sarpong N, Ekuban S et al. (2010) Typhoid fever among children, Ghana. Emerg Infect Dis 16: 1796-1797. doi:10.3201/eid1611.100388. PubMed: 21029549. 21029549 PMC3294512

[11] Krumkamp R, Schwarz NG, Sarpong N, Loag W, Zeeb H et al. (2012) Extrapolating respiratory tract infection incidences to a rural area of Ghana using a probability model for hospital attendance. Int J Infect Dis 16: e429-e435. doi:10.1016/j.ijid.2012.05.601. PubMed: 22484157. 22484157

[12] Nielsen MV, Sarpong N, Krumkamp R, Dekker D, Loag W et al. (2012) Incidence and Characteristics of Bacteremia among Children in Rural Ghana. PLOS ONE 7: e44063. doi:10.1371/journal.pone.0044063. PubMed: 22970162. 22970162 PMC3438186

[13] Sarpong N, Loag W, Fobil J, Meyer CG, Adu-Sarkodie Y et al. (2010) National health insurance coverage and socio-economic status in a rural district of Ghana. Trop Med Int Health 15: 191-197. doi:10.1111/j.1365-3156.2009.02439.x. PubMed: 19961565. 19961565

[14] Vyas S, Kumaranayake L (2006) Constructing socio-economic status indices: how to use principal components analysis. Health Policy Plan 21: 459-468. doi:10.1093/heapol/czl029. PubMed: 17030551. 17030551

[15] Zou G (2004) A modified poisson regression approach to prospective studies with binary data. Am J Epidemiol 159: 702-706. doi:10.1093/aje/kwh090. PubMed: 15033648. 15033648

[16] Greenland S (2004) Model-based estimation of relative risks and other epidemiologic measures in studies of common outcomes and in case-control studies. Am J Epidemiol 160: 301-305. doi:10.1093/aje/kwh221. PubMed: 15286014. 15286014

[17] Feikin DR, Nguyen LM, Adazu K, Ombok M, Audi A et al. (2009) The impact of distance of residence from a peripheral health facility on pediatric health utilisation in rural western Kenya. 14: 54-61. 10.1111/j.1365-3156.2008.02193.x 19021892

[18] NoorAli R, Luby S, Rahbar MH (1999) Does use of a government service depend on distance from the health facility? Health Policy Plan 14: 191-197. doi:10.1093/heapol/14.2.191. PubMed: 10538722. 10538722

[19] Bigogo G, Audi A, Aura B, Aol G, Breiman RF et al. (2010) Health-seeking patterns among participants of population-based morbidity surveillance in rural western Kenya: implications for calculating disease rates. Int J Infect Dis 14: e967-e973. doi:10.1016/j.ijid.2010.05.016. PubMed: 20800525. 20800525

[20] Alegana VA, Wright JA, Pentrina U, Noor AM, Snow RW et al. (2012) Spatial modelling of healthcare utilisation for treatment of fever in Namibia. Int J Health Geogr 11: 6. doi:10.1186/1476-072X-11-6. PubMed: 22336441. 22336441 PMC3292929

[21] Okeke TA, Okeibunor JC (2010) Rural-urban differences in health-seeking for the treatment of childhood malaria in south-east Nigeria. Health Policy 95: 62-68. doi:10.1016/j.healthpol.2009.11.005. PubMed: 20004038. 20004038

[22] Okwaraji YB, Cousens S, Berhane Y, Mulholland K, Edmond K (2012) Effect of geographical access to health facilities on child mortality in rural Ethiopia: a community based cross sectional study. PLOS ONE 7: e33564. doi:10.1371/journal.pone.0033564. PubMed: 22428070. 22428070 PMC3299799

[23] Schoeps A, Gabrysch S, Niamba L, Sié A, Becher H (2011) The effect of distance to health-care facilities on childhood mortality in rural Burkina Faso. Am J Epidemiol 173: 492-498. doi:10.1093/aje/kwq386. PubMed: 21262911. 21262911

[24] Kazembe LN, Appleton CC, Kleinschmidt I (2007) Choice of treatment for fever at household level in Malawi: examining spatial patterns. Malar J 6: 40. doi:10.1186/1475-2875-6-40. PubMed: 17425775. 17425775 PMC1855348

[25] Bhatti MA (2005) Geogr Patterns Access Utilization Basic Health Units District. Attock: 48–53.

[26] Schellenberg JA, Victora CG, Mushi A, de Savigny D, Schellenberg D et al. (2003) Inequities among the very poor: health care for children in rural southern Tanzania. Lancet 361: 561-566. doi:10.1016/S0140-6736(03)12515-9. PubMed: 12598141. 12598141

[27] van der Hoeven M, Kruger A, Greeff M (2012) Differences in health care seeking behaviour between rural and urban communities in South Africa. Int J Equity Health 11: 31. doi:10.1186/1475-9276-11-31. PubMed: 22691443. 22691443 PMC3419677

[28] Rutebemberwa E, Kallander K, Tomson G, Peterson S, Pariyo G (2009) Determinants of delay in care-seeking for febrile children in eastern Uganda. Trop Med Int Health 14: 472-479. doi:10.1111/j.1365-3156.2009.02237.x. PubMed: 19222823. 19222823

[29] Danso-Appiah A, Stolk WA, Bosompem KM, Otchere J, Looman CW et al. (2010) Health seeking behaviour and utilization of health facilities for schistosomiasis-related symptoms in ghana. PLoS Negl Trop. Drosophila Inf Serv 4: e867. 10.1371/journal.pntd.0000867 PMC2970540 21072229

[30] Onwujekwe O (2005) Inequities in healthcare seeking in the treatment of communicable endemic diseases in Southeast Nigeria. Soc Sci Med 61: 455-463. doi:10.1016/j.socscimed.2004.11.066. PubMed: 15893059. 15893059

[31] Onwujekwe O, Hanson K, Uzochukwu B (2011) Do poor people use poor quality providers? Evidence from the treatment of presumptive malaria in Nigeria. Trop Med Int Health 16: 1087-1098. doi:10.1111/j.1365-3156.2011.02821.x. PubMed: 21702870. 21702870

[32] Mills A, Ataguba JE, Akazili J, Borghi J, Garshong B et al. (2012) Equity in financing and use of health care in Ghana, South Africa, and Tanzania: implications for paths to universal coverage. Lancet 380: 126-133. doi:10.1016/S0140-6736(12)60357-2. PubMed: 22591542. 22591542

[33] McIntyre D, Garshong B, Mtei G, Meheus F, Thiede M et al. (2008) Beyond fragmentation and towards universal coverage: insights from Ghana, South Africa and the United Republic of Tanzania. Bull World Health Organ 86: 871-876. doi:10.2471/BLT.08.053413. PubMed: 19030693. 19030693 PMC2649570

[34] Macha J, Harris B, Garshong B, Ataguba JE, Akazili J et al. (2012) Factors influencing the burden of health care financing and the distribution of health care benefits in Ghana, Tanzania and South Africa. Health Policy Plan 27 Suppl 1: i46-i54. doi:10.1093/heapol/czs024. PubMed: 22388500. 22388500

[35] Nonvignon J, Aikins MK, Chinbuah MA, Abbey M, Gyapong M et al. (2010) Treatment choices for fevers in children under-five years in a rural Ghanaian district. Malar J 9: 188. doi:10.1186/1475-2875-9-188. PubMed: 20584280. 20584280 PMC2914057

[36] Taffa N, Chepngeno G (2005) Determinants of health care seeking for childhood illnesses in Nairobi slums. Trop Med Int Health 10: 240-245. doi:10.1111/j.1365-3156.2004.01381.x. PubMed: 15730508. 15730508

[37] Tinuade O, Iyabo RA, Durotoye O (2010) Health-care-seeking behaviour for childhood illnesses in a resource-poor setting. J Paediatr Child Health 46: 238-242. doi:10.1111/j.1440-1754.2009.01677.x. PubMed: 20337870. 20337870

[38] Greenland S, Thomas DC (1982) On the need for the rare disease assumption in case-control studies. Am J Epidemiol 116: 547-553. PubMed: 7124721. 7124721 10.1093/oxfordjournals.aje.a113439

[39] Carrera C, Azrack A, Begkoyian G, Pfaffmann J, Ribaira E et al. (2012) The comparative cost-effectiveness of an equity-focused approach to child survival, health, and nutrition: a modelling approach. Lancet 380: 1341-1351. doi:10.1016/S0140-6736(12)61378-6. PubMed: 22999434. 22999434

